# Effect of chitosan nanoparticles on quorum sensing-controlled virulence factors and expression of *LasI* and *RhlI* genes among *Pseudomonas aeruginosa* clinical isolates

**DOI:** 10.3934/microbiol.2021025

**Published:** 2021-10-26

**Authors:** Rana Abdel Fattah Abdel Fattah, Fatma El zaharaa Youssef Fathy, Tahany Abdel Hamed Mohamed, Marwa Shabban Elsayed

**Affiliations:** Department of Medical Microbiology and Immunology, Faculty of Medicine, Ain Shams University, Cairo, Egypt

**Keywords:** Chitosan nanoparticles, quorum sensing-controlled virulence factors, expression of *LasI* and *RhlI* genes, *Pseudomonas aeruginosa*

## Abstract

Antibiotic-resistant strains of *Pseudomonas aeruginosa (P. aeruginosa*) pose a major threat for healthcare-associated and community-acquired infections. *P. aeruginosa* is recognized as an opportunistic pathogen using quorum sensing (QS) system to regulate the expression of virulence factors and biofilm development. Thus, meddling with the QS system would give alternate methods of controlling the pathogenicity. This study aimed to assess the inhibitory impact of chitosan nanoparticles (CS-NPs) on *P. aeruginosa* virulence factors regulated by QS (e.g., motility and biofilm formation) and *LasI* and *RhlI* gene expression. Minimum inhibitory concentration (MIC) of CS-NPs against 30 isolates of *P. aeruginosa* was determined. The CS-NPs at sub-MIC were utilized to assess their inhibitory effect on motility, biofilm formation, and the expression levels of *LasI* and *RhlI* genes. CS-NPs remarkably inhibited the tested virulence factors as compared to the controls grown without the nanoparticles. The mean (±SD) diameter of swimming motility was decreased from 3.93 (±1.5) to 1.63 (±1.02) cm, and the mean of the swarming motility was reduced from 3.5 (±1.6) to 1.9 (±1.07) cm. All isolates became non-biofilm producers, and the mean percentage rate of biofilm inhibition was 84.95% (±6.18). Quantitative real-time PCR affirmed the opposition of QS activity by lowering the expression levels of *LasI* and *RhlI* genes; the expression level was decreased by 90- and 100-folds, respectively. In conclusion, the application of CS-NPs reduces the virulence factors significantly at both genotypic and phenotypic levels. These promising results can breathe hope in the fight against resistant *P. aeruginosa* by repressing its QS-regulated virulence factors.

## 1. Introduction

*Pseudomonas aeruginosa (P. aeruginosa)* is an opportunistic Gram-negative bacterium that contributes significantly to healthcare-associated infections. The CDC has reported it as a major lead causative agent of pneumonia, the third cause of urinary tract infection, eighth commonly isolated microbe from bloodstream infections. Additionally, it is responsible for fatal infections in cystic fibrosis and immunocompromised patients [1].

The success of *P. aeruginosa*, as an opportunistic pathogen, is partially attributed to the ability of whole bacterial populations of this bacterium to coordinate their activity using cell-to-cell communication, mediated by quorum sensing (QS) signal molecules [2]. QS regulates over 10% of the *P. aeruginosa* genome, including swarming motility, biofilm formation, and antimicrobial resistance, as well as the production of virulence determinants such as elastases, pyocyanin, cyanide, and exotoxins [3].

*P.aeruginosa* employs two dominating QS systems; *las* (*LasR-LasI*) and *rhl* (*RhlR-RhlI*). The synthase of *LasI* catalyzes the synthesis of N-(3-oxododecanoyl) homoserine lactone, *RhlI* catalyzes the synthesis of N-butyryl-homoserine lactone, which induces their respective cognate transcriptional regulators. *LasR* and *RhlR* are responsible for activating numerous QS-controlled genes [4].

The rise of drug-resistant *P. aeruginosa* and the delay in introducing newer drugs threaten human well-being. As a better drug strategy, it has been suggested to focus on QS-regulated virulence factors instead of growth-related features [5]. Among the most accepted materials is chitosan, a polysaccharide polymer composed of N-acetyl-D-glucosamine and D-glucosamine units connected by β-1,4-glycosidic linkages. It is derived from partial or total deacetylation of chitin [6]. It possesses a unique combination of properties, mainly excellent biocompatibility, enzymatic biodegradability, metal complexation, and nontoxicity [7]. It has a good antimicrobial action against a wide scope of microorganisms such as *Staphylococcus aureus* and *Escherichia coli*; also, it has an antifungal effect [8],[9].

Advances in nanotechnology have enabled the formulation of chitosan polymers as nanoparticles, which are more effective antimicrobial agents than chitosan itself, in addition to other benefits, such as better penetration of biofilm and higher solubility [10]. This study aimed to assess the inhibitory impact of chitosan nanoparticles (CS-NPs) on virulence characters of *P. aeruginosa* regulated by QS as motility and biofilm formation and the expression of *LasI* and *RhlI* genes to find new alternatives to the existing antibiotic therapy for treating resistant infections.

## 2. Materials and methods

The present study was conducted from October 2020 till February 2021 on 30 *P. aeruginosa* isolates obtained from inpatient and outpatient clinical samples submitted to the central microbiology laboratory at Ain Shams University Hospital. The Research Ethics Committee approved the study of Faculty of Medicine Ain Shams University (No. FMASU M D 94/2020).

The isolates included sputum, blood, urine, and swabs from surgical and burned wounds, collected under complete aseptic precautions, identified by conventional microbiologic methods [11]. All *P. aeruginosa* isolates were stored at −80 °C in nutrient broth with 20% (vol/vol) glycerol.

### 2.1. Antimicrobial susceptibility testing

According to Clinical and Laboratory Standards Institute guidelines, antimicrobial susceptibility testing was done for the 30 *P. aeruginosa* clinical isolates using the disk diffusion method [12]. The clinical isolates were tested for their susceptibility to the following antibiotics *(Oxoid, England)*: aztreonam (30 µg), cefepime (30 µg), ceftazidime (30 µg), ciprofloxacin (5 µg), fosfomycin (200 µg), gentamicin (10 µg), tobramycin (10 µg), amikacin (30 µg), meropenem (10 µg), and piperacillin/Tazobactam (100 µg). MDR organism was defined as the isolate was non-susceptible to at least one drug in three or more classes of antimicrobial [13].

### 2.2. Preparation of CS-NPs

Chitosan nanoparticles with a molecular weight less than 100 kDa, 85% degree of deacetylation, size less than 50nm, spherical in shape, and concentration 20 mg/mL were purchased from nanogate *(www.nanogate-eg.com)*. The manufacturer prepared CS-NPs according to the ionotropic gelation process [14]. Blank nanoparticles were obtained upon the addition of a tripolyphosphate aqueous solution to a chitosan solution. Transmission Electron Microscopy was performed on JEOL JEM-2100 high-resolution transmission electron microscope at an accelerating voltage of 200 kV, respectively.

### 2.3. Determination of the minimum inhibitory concentration (MIC) of CS-NPs

The broth microdilution method was used to determine the MIC of CS-NPs [12]. Two-fold serial dilutions of CS-NPs in Muller-Hinton broth *(Oxoid, UK)* were prepared to reach a final volume of 0.1 mL of each concentration in each well. A standardized inoculum using the direct colony suspension was prepared and diluted in sterile saline to obtain the suspension's final concentration of 0.5 McFarland. Then the inoculum was diluted at 1:20 to yield 5 × 10^6^ CFU/mL. Finally, 0.01 mL of this suspension was added to each well. Negative control tubes containing broth only and other negative control tubes containing CS-NPs only with different concentrations. The microtiter plate was incubated at 37 °C for 24 hrs.

**Figure 1.**
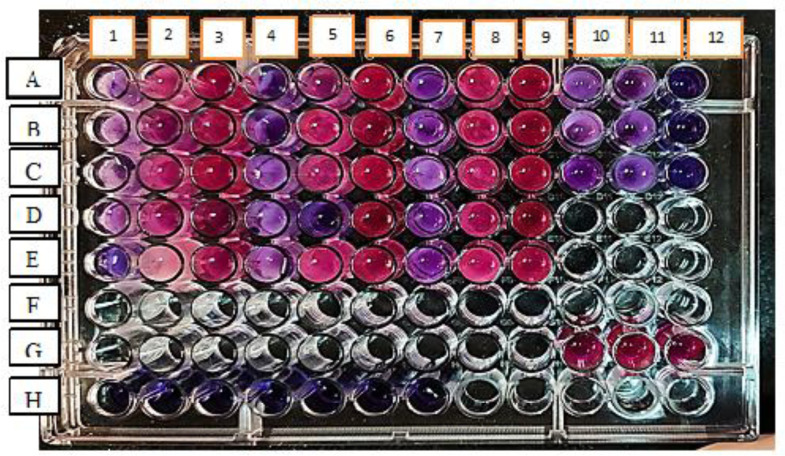
MIC determination of CS-NPs against *P. aeruginosa* isolates. Each row of A, B, C, D, E contained one isolate tested with three different concentrations of CN-NPs. Each isolate was tested in triplicate. Row H confirmed no contamination occurred while preparing the plate as no change of resazurin natural color (blue/purple) to the reduced form (red-colorless). Row G (columns 10, 11, 12) were positive controls contained bacterial suspension only. Row A (columns 10, 11, 12) contained only CS-NPs with different concentrations. The highest concentration incorporated into the plate was 20 mg/mL, and the lowest achieved through double serial dilution was 5 mg/mL. Columns 1, 4, 7 showed no color changes; therefore, concentration in these columns was taken as the MIC value. Columns 2, 3, 5, 6, 8, 9 showed color change; therefore, the isolates were viable.

The MIC of CS-NPs against *P. aeruginosa* clinical isolates was determined using the resazurin microtiter plate assay[15]. This assay uses the redox indicator resazurin (*Sigma-Aldrich, Germany*) that changed color from blue to pink in the presence of viable cells. The MIC was determined as the concentration at which there was no color change following 4 hrs incubation of the overnight cells with 0.015% resazurin (Figure 1). One dilution below the MIC was regarded as the sub-MIC concentration and was used to evaluate the ability of the CS-NPs to inhibit the virulence activity.

### 2.4. Evaluation of two virulence factors of P. aeruginosa and the phenotypic inhibitory activity of CS-NPs on these virulence factors

#### 2.4.1. Motility assay

*P. aeruginosa* can swim on soft surfaces, twitch on hard surfaces, and swarm on semi-solid surfaces. An overnight culture of isolates was diluted to 0.5 McFarland. Flagellum-dependent swimming was performed using swimming media. Swimming media composed of 8.0 g bacteriological agar, 5.0 g NaCl, 10.0 g Tryptone per liter, PH 7.0 ± 0.2 at 25 °C. While swarming motility was assayed using swarming media, Swarming media composed of 5.0 g agar, 10.0 g glucose, 5.0 g peptone, 2.0 g yeast extract per liter, PH 7.0 ± 0.2 at 25 °C *(Lab M Limited, UK)*. The plates were inoculated with 2 µL of diluted (0.5 McFarland) culture then incubated for 24 hours (hrs) and 48 hrs at 37 °C. The diameter of the turbid zone (mm) was measured [16]. The assay was performed in triplicate, and the mean of the diameter was assigned.

##### 2.4.1.1. The phenotypic inhibitory activity of CS-NPs on motility

The inhibitory effect of CS-NPs on motility was performed using the previously described method with the addition of sub-MICs of CS-NPs.

#### 2.4.2. Quantitative detection of biofilm formation by microtiter plate crystal violet assay

A loopful of overnight cultures of the tested organisms was inoculated into 5mL of Tryptic soy broth (TSB) (*Lab M Limited, UK*) with 1% glucose and incubated at 37 °C for 24 hrs. Each well of sterile 96 well-flat bottom polystyrene tissue culture plate *(Sigma-Aldrich Co. LLC, USA)* was filled with 200 uL of the bacterial suspension corresponding to 0.5 McFarland after further dilution of 1:100 with fresh medium along with control organisms. Only broth was served as a negative control to check the sterility and non-specific binding of media. The plates were incubated at 37 °C for 24 hrs. After incubation, contents of each well were removed by gentle tapping and wells were washed three times with 300 µL of sterile saline. The remaining attached bacteria were heat-fixed by exposing them to hot air at 60 °C for 60 min. Then 150 µL of crystal violet stain was added to each well. After 15 min, the excess dye was rinsed off by decantation, and the plate was washed. About 150 µL of 95% ethanol was added to each well. After 30 min, the optical densities (OD) of stained adherent bacterial films were read using a microtiter plate reader at 492 nm and 630 nm.

The test was carried out in triplicate, and the results were averaged. The OD values were calculated for all tested strains and negative controls, the cut-off value (ODc) was established. For interpretation of the results, strains were divided into the following categories: non-biofilm producer (0): OD ≤ ODc, weak biofilm producer (1+): ODc < OD ≤ 2 × ODc, moderate biofilm producer (2+): 2 × ODc < OD ≤ 4 × ODc, strong biofilm producer (3+): 4 × ODc < OD [17],[18].

##### 2.4.2.1. Phenotypic inhibitory activity of CS-NPs on biofilm formation

The inhibitory effect of CS-NPs on ofilm formation was performed using the previously described method with the addition of sub-MICs of CS-NPs. The inhibition of biofilm formation was calculated using the equation below.

$$\text{Inhibition rate}=1-\frac{OD\;Treatment}{OD\;Control}\times 100$$

[19]

### 2.5. Genotypic detection of inhibitory activity of CS-NPs by Quantitative Real-Time PCR

The most virulent *P. aeruginosa* clinical isolates were cultivated in Luria Bertani medium *(OXOID, UK)* supplemented with sub- MICs of CS-NPs until (18 hrs); the middle of the exponential growth phase (OD 600; 0.5 McFarland). The total RNA was extracted using Gene JET RNA Purification Kit according to the manufacturer's instructions. According to the manufacturer's instructions, the complementary DNA was synthesized using Thermo Scientific Verso SYBR Green 1-Step QRT-PCR Kit Plus ROX Vial (Thermo Scientific, Lithuania). Quantitative Real-Time PCR was used to measure the effect of CS-NPs on the expression of QS genes *LasI* and *RhlI* in treated and untreated cultures, in duplicates using the following primers *RopD* forward, 5-CGAACTGCTTGCCGACTT-3 and *RopD* reverse, 5-GCGAGAGCCTCAAGGATAC-3; *LasI* forward, 5-CGCACATCTGGGAACTCA-3 and *LasI* reverse, 5-CGGCACGGATCATCATCT-3; *RhlI* forward 5-GTAGCGGGTTTGCGGATG-3 and *RhlI* reverse, 5-CGGCATCAGGTCTTCATCG-3. The reaction mixture was prepared as in the manufacturer's instructions. Then the thermal cycling was programmed as follows; cDNA synthesis at 50 °C for 15 min 1 cycle, Thermo-Start activation at 95 °C for 15 min 1 cycle, 40 cycles of denaturation at 95 °C for 15 sec, annealing at 50 °C–60 °C for 30 sec, and extension at 72 °C for 30 sec. The reaction volume was set to 25 µL, then loaded into the thermal cycler then the reverse transcription run started. Melt curve was performed to confirm the specificity of the reaction. The expressions of the quantified genes were normalized to the expression of the housekeeping gene *RopD* because no change in the expression levels of this gene in treated and untreated cultures is exhibited. The gene expression level of treated *P. aeruginosa* was calculated relative to that in the untreated *P. aeruginosa* using 2−ΔΔCt method [20].

### 2.6. Statistical analysis

Analysis of the results was done using SPSS version 22. Quantitative data were expressed in the form of mean and standard deviation or median and range as appropriate. Paired t-test was utilized to study the significance of the inhibitory activity of CS-NPs on the motility of *P. aeruginosa* clinical isolates. Willcoxon Rank test was used to investigate the significance of the inhibitory activity against biofilm formation and expression of QS genes *LasI* and *RhlI*. P-values < 0.05 were statistically significant.

## 3. Results

The *P. aeruginosa* strains enrolled in this study were obtained from different clinical samples. Most of isolates were from urine samples (13/30, 43.3%) followed by sputum (7/30, 23.3%), pus (6/30, 20%), CSF and blood (each 2/30, 6.6%). The antibiotic sensitivity pattern of isolated strains showed that they were highly resistant to cefepime, ceftazidime, and gentamycin with rates of 96%, 90%, and 87%, respectively. About 80% (24/30) of the isolates were MDR.

**Figure 2.**
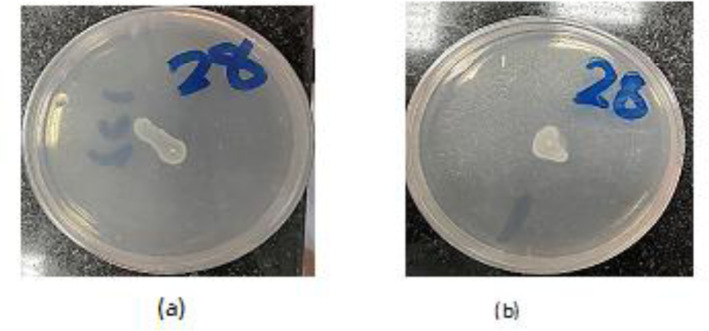
(a) Swimming motility of *P. aeruginosa isolate*. (b) The effect of sub-MIC concentrations of CS-NPs on swimming motility of these isolate.

**Figure 3.**
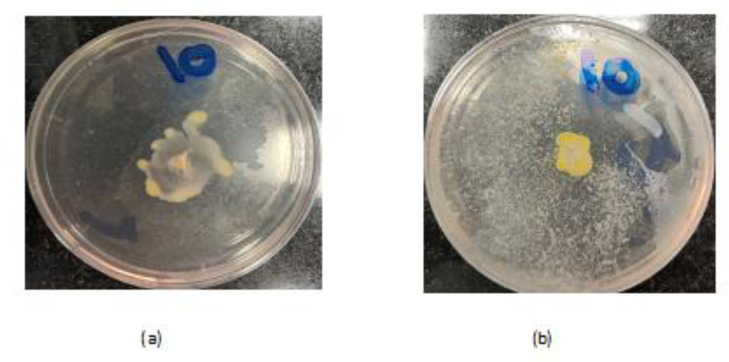
(a) Swarming motility of *P. aeruginosa isolates*, (b) The effect of sub-MIC concentrations of CS-NPs on swarming motility of these isolates.

All 30 *P. aeruginosa* isolates exhibited both swimming and swarming motility (Figure 2a and 3a). The inhibitory activity of CS-NPs on the motility of clinical isolates was assessed at the sub-MICs (it differed among the strains, but it ranged from 5 mg/mL to 10 mg/mL) and is shown in (Figures 2b and 3b). The CS-NPs reduced the swimming and swarming motility of all bacterial isolates. The test differed from the control with a P-value of 0.0001. The mean (±SD) diameter of swimming motility was decreased from 3.93 (±1.5) to 1.63 (±1.02) cm, and the mean of the swarming motility was reduced from 3.5 (±1.6) to 1.9 (±1.07) cm (Table 1).

**Table1.** Effect of chitosan nanoparticles on motility and biofilm formation of *P. aeruginosa* isolates.

Isolate number	Swimming motility (diameter in cm)		Swarming motility (diameter in cm)		Biofilm formation (Absorbance)		
	Control	Chitosan nanoparticles	Control	Chitosan nanoparticles	Control	Chitosan nanoparticles	Rate of biofilm inhibition (%)
8	4.5	2	3	1	0.587 (+2)	0.063 (0)	89.2
9	5	1.8	5	1.7	0.664 (+2)	0.069 (0)	89.6
10	3.5	1	3	1.3	0.792 (+2)	0.01 (0)	98.7
11	5.5	2	2	2	0.722 (+2)	0.19 (0)	73.6
16	4.2	2.1	3	2.2	0.593 (+2)	0.116 (0)	80.4
17	5	3	4.5	2.5	0.686 (+2)	0.07 (0)	89.7
20	5	4.8	4	1.5	0.661 (+2)	0.081 (0)	87.7
22	7	4	5	4	0.695 (+2)	0.179 (0)	74.2
23	3.5	0.8	4.5	1	0.545 (+1)	0.044 (0)	91.9
25	3	1	5	1	0.635 (+2)	0.058 (0)	90.8
26	5	1	7	4	0.728 (+2)	0.163 (0)	77.6
27	3.6	0.5	3.5	2	0.739 (+2)	0.079 (0)	89.3
28	4.8	1.3	1.3	1.3	0.692 (+2)	0.114 (0)	83.5
29	3.5	0.5	1	0.8	0.526 (+1)	0.128 (0)	75.6
30	4.5	1.25	2	0.5	0.713 (+2)	0.128 (0)	82
Mean (n = 30)	3.93	1.63	3.5	1.9	0.667	0.099	84.95
±SD	1.5	1.02	1.6	1.07	0.096	0.045	6.18

**Figure 4.**
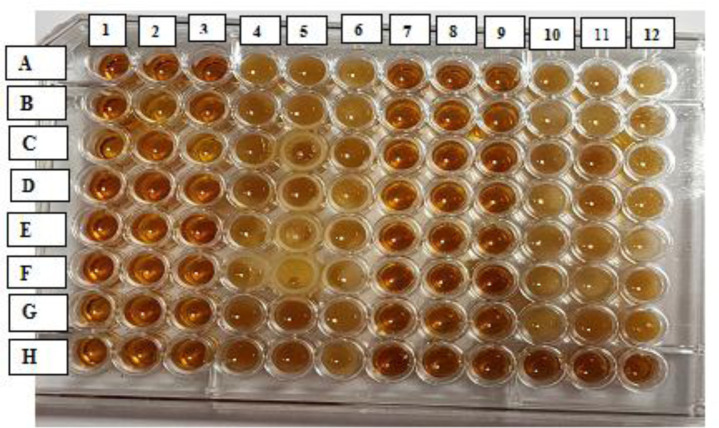
Microtiter plate assay for assessment of biofilm formation by *P. aeruginosa* isolates. Each isolate was tested in triplicate, without and with the addition of sub-MIC of CS-NPs. Each row contained two isolates. Row A: showed two isolates, columns 1, 2, 3 contained isolate no.1 without CS-NPs and columns 4,5,6 contained the same isolate no.1 in addition to sub-MIC of CS-NPs, and isolate no.2 in columns (7,8,9), so on till row H (column 6). Row H (columns 7, 8, 9, 10, 11, 12) contained tryptone soya broth only.

**Figure 5.**
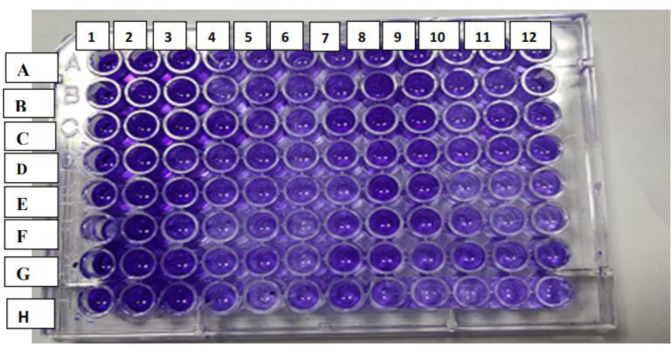
Quantitative crystal violet microtiter plate assay showed the effect of sub-MIC of CS-NPs on the biofilm formation of *P. aeruginosa* isolates. Columns 1, 2, 3, 7, 8, 9 showed that the isolates were more strongly stained, indicating stronger ability to form biofilm mass. While isolates in columns 4, 5, 6, 10, 11, 12 showed a decrease in staining intensity, indicating decreasing biofilm formation by CS-NPs. Row (H) columns 7, 8, 9, 10, 11, 12 were negative controls.

All 30 *P. aeruginosa* isolates showed biofilm production by microtiter plate assay. About 83.3% (25/30) of the isolates were moderate biofilm producers (+2), and 16.7% (5/30) were weak biofilm producers (+1). CS-NPs with the sub-MICs significantly reduced the biofilm formation by *P. aeruginosa* isolates (p-value < 0.01) as 100% of isolates became a non-biofilm producer (0) (Figures 4 and 5). The mean (±SD) of OD was decreased from 0.667 (±0.096) to 0.099 (±0.045). The mean percentage rate of biofilm inhibition was 84.95% (±6.18) (Table 1).

**Table 2.** Effect of CS-NPs on levels of *LasI* and *RhlI* expression in isolated *P. aeruginosa* strains.

Isolate number	*Las I* cycle threshold		The fold of decrease of *LasI* expression	*RhlI* cycle threshold		The fold of decrease of *Rhl I* expression
	Control	Chitosan nanoparticles		Control	Chitosan nanoparticles	
8	15.94	25.2	500	17.42	27.3	1000
9	10.4	22.6	1000	11.63	23.01	625
10	13.6	20.9	34.48	14.6	21.8	32.25
11	12	22	256	13	21	64
16	10	12.03	5	10.86	12.6	9.25
17	11.5	13.5	16	12	14	16
20	16.1	24.63	64.1	15.88	25.01	128
22	11.5	13.5	4	12.5	14.9	6.5
23	15.3	26	416	15.9	23.8	64
25	11.5	26	90	10.8	25	100
26	13	23	32	11	24	256
27	11.8	21.4	400	13.08	21.8	250
28	23.7	30.5	16.6	12.22	19.7	66.6
29	12.5	29.5	3333.3	11.5	26.5	1176
30	13.3	25.2	4166	11.75	20.1	200
Median (n = 15)	12.5	23	90	12.22	21.8	100
Mean	13.5	22.4	689	13	21.4	266
±SD	3.4	5.6	1282	2	4.5	370

**Figure 6.**
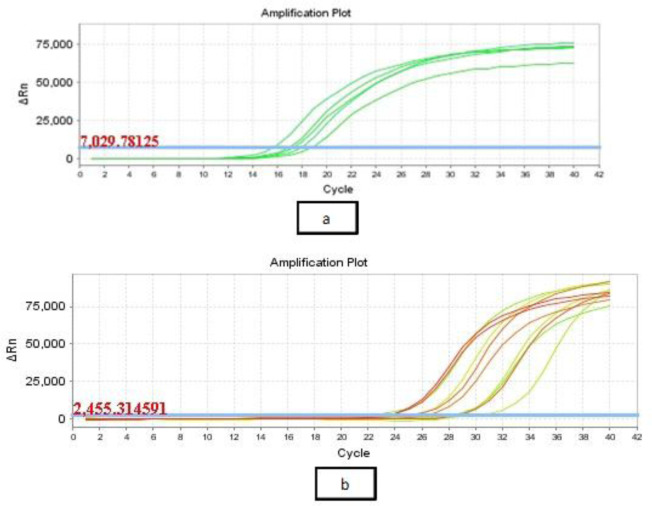
The cycle threshold of *Las I* in *P. aeruginosa* isolates (a) before and (b) after treatment with the sub-MICs of CS-NPs.

**Figure 7.**
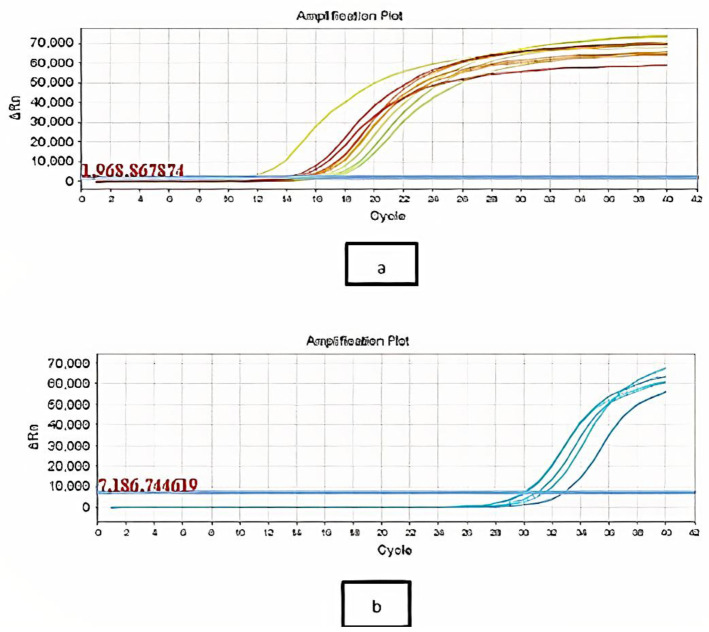
The cycle threshold of *RhlI* in *P. aeruginosa* isolates (a) before and (b) after treatment with the sub-MICs of CS-NPs.

CS-NPs inhibited virulence activity on the genotypic level as well (Figures 6 and 7). At the sub-MIC concentration of CS-NPs, the median of *Las I* cycle threshold (CT) was increased from 12.5 to 23, and the median CT of *RhlI* was increased from 12.22 to 21.8. The levels of *LasI* and *RhlI* expression were significantly lowered compared with untreated cultures. The median fold decrease of *LasI*, and *RhlI* expression in selected most virulent 15 isolates was 90 and 100, respectively (Table 2). The inhibition of gene expression was expressed by median due to the high variability of inhibition values ranging from 4166 to 5 and 1176 to 6.5 in *Las I* and *RhlI*, respectively, upon treatment with CS-NPs.

## 4. Discussion

*P. aeruginosa* is an opportunistic microbe that can produce different types of infection, especially in immunocompromised patients [21]. The QS network controls bacterial virulence and biofilm-forming ability. It includes transcriptional regulators such as Las and Rhl, activated by their natural autoinducers [22].

Arise of MDR strains of *P. aeruginosa* is an expanding issue worldwide that messes up therapy of infections, resulting in remarkable morbidity and mortality rates [23]. In the current study, *P. aeruginosa* isolates exhibited high resistance rates to cefepime, ceftazidime, gentamicin, ciprofloxacin, and tobramycin. About 80% of the isolates were MDR. *Pe et al*. [24] reported a nearly similar rate of drug resistance. In contrast, a lower rate was reported by *Sala et al*. [25]. The antimicrobial susceptibility pattern varies among hospitals and populations, which can be explained by lack of antibiotic policy, non-compliance of patients, and non-adherence to infection control measures [26].

The latest reports have assessed new methods to fight against *P. aeruginosa* virulence factors that rely on QS inhibition [27]. The QS inhibitors restrain several virulence factors and biofilm without influencing growth, unlike antibiotics, thus could permit better function of the individual's immunity and externally administered antibiotics [1]. Subsequently, this approach is by all accounts promising to address these MDR pathogens and can be used as independent anti-infective drugs or in combination with other traditional antibiotics [28].

CS-NPs displayed significant antibacterial activity against *K. pneumoniae, E. coli, S. aureus, and P. aeruginosa*. This activity is more prominent in comparison to chitin and chitosan [8]. Various speculations have clarified the antibacterial activity of CS-NPs; the most widely recognized is the electrostatic communication between the positively charged amino groups on chitosan and the negative charges on the bacterial surface. An impermeable layer is formed around the cell that blocks the transport of important solutes through the outer membrane of Gram-negative bacteria. Also, this interaction alters the structure and permeability of cell membrane resulting in leakage of intracellular constituents [29],[30], followed by attachment to DNA hindering its replication that ends in cell death [31],[32]. Another mechanism is that chitosan chelates trace metal elements, causing toxin production and inhibiting microbial growth [10].

Thus, CS-NPs have become an expected possibility for combatting this era of multi-drug resistance. Past work by *Aleanizy et al*. [33] reported the remarkable antimicrobial impact of CS-NPs against *P. aeruginosa*. Their work gave proof of the anti-virulence activities of CS-NPs. In the current study, we focused on the inhibitory effect of CS-NPs on phenotypic virulence factors of *P.aeruginosa* regulated by QS as motility and biofilm formation. We confirmed our results on the genotypic level by performing Quantitative Real-Time PCR to detect the fold of decrease in expression of QS regulated *LasI and RhlI* genes.

Bacterial motility relates to its capacity of tissue adherence and biofilm formation and is under the control of QS systems as *LasI/R* and *RhlI/R* [34]. Motility enables the bacteria by giving nutrients, locomotion towards the substrate or host, getting away from the antibiotics, and spreading the biofilm [35]. In addition, it was observed that strains with impaired motilities could only form thin-spreading biofilms [36]. In the current study, the mean diameter of swimming motility in untreated cultures was 3.93 (±1.5), while that of swarming motility was 3.5 (±1.6) cm. However, in the presence of CS-NPs, the mean diameter of swimming motility was reduced to 1.63 (±1.2) cm, and that of swarming motility was decreased to 1.9(±1.07) cm. This almost concurs with past work by *Khan et al*. [37], they noticed a remarkable reduction in the diameter of the swimming motility zone from 3.05 (±0.07) cm to 0.95 (±0.07) cm in the presence of chitosan-polypyrrole nanocomposites, the diameter of swarming motility was reduced from 2.6 (±0.21) cm to 1.5 (±0.14). However, the bacterial isolates in their study were standard control strains, not strains isolated from clinical samples. There was no confirmation of the results by genotypic methods. *Rubini et al*. [38] also showed that chitosan had significant action in hindering the swarming motility of the tested strains. The authors evaluated the inhibitory effect of chitosan, not CS-NPs, on standard strains. Along the same line, *Badawy et al*. [17] tracked down that treatment with CH/ZnO nanocomposite reduced both swimming and swarming motility from 5.9 (±0.7) and 5.6 (±1.0) to 2.7 (±1.5) and 3.2 (±0.9) mm, respectively. They conducted their tests on only 10 most virulent isolates of *P.aeruginosa* and standard control strain PAO1.

Besides regulating virulence characters, QS controls *P. aeruginosa* biofilm-forming ability. A study showed that *P. aeruginosa* with mutations in *lasR* and *lasI* form impaired biofilms that are easily eliminated by antimicrobials [39]. Genes activated by QS encode exopolysaccharides and other items that control the structure of the developing communities [40]. The inhibitory action of chitosan on biofilm development was documented in several studies. *Divya et al*. [19] reported that chitosan has antibiofilm action against *P. aeruginosa, E.coli*, *Staphylococcus aureus*, and *klebsiella pneumoniae* with an inhibition rate up to 85%, 97%, 98%, and 94%, respectively. The study carried out by *Divya et al*. focused on the phenotypic inhibitory effect of CS-NPs on standard strains without confirming their results by genotypic methods. *Muslim et al*. [41] noticed a huge decline in biofilm development in the presence of chitosan, obviously confirmed under both light and scanning electron microscopy. Their study evaluated chitosan instead of CS-NPs, and the tests were carried out on standard strain. Some authors reported that both extracted and commercial chitosan of sub-MIC strongly inhibited *P. aeruginosa* and *Serretia marcescens* biofilms, with a maximum degree of inhibition (58–65%) and p-value significance of <0.001 [38]. In the current study, treatment of *P. aeruginosa* isolates with CS-NPs suppressed biofilm formation as mean OD decreased from 0.667 (±0.096) to 0.099 (±0.045). The rate of inhibition of biofilm formation was 84.95% (±6.18) among the thirty isolates. Such inhibitory activity may be related to inhibiting exopolysaccharide synthesis or disseminating CS-NPs through the biofilm channels [41],[42].

Most studies investigating QS and its role in *P. aeruginosa* pathogenicity have highlighted the *LasI/R* system because of its position at the head of the QS signal transduction pathway. Accordingly, any changes in their expression would influence the phenotype of the organism [43]. In the current study, we found a remarkable increase in *Las I* gene's mean cycle threshold from 12.5 to 23 and *RhlI* gene from 12.22 to 21.8 after treatment by CS-NPs. The expression of *Las I* and *RhlI* was decreased by 90 and 100 folds, respectively. Previous work by *Muslim et al*. [41] showed a reduction in the expression level of *LasR* and *RhlR* genes by 32 and 8 folds, respectively. *Rubini et al*. [38] reported that both extracted chitosan and commercial chitosan exhibited 0.2–0.4 fold down-regulation of *LasI* and *RhlI* expression levels in the exposed *P. aeruginosa* isolates. *Badawy et al*. [17] announced that the chitosan decreased *LasI* and *RhlI* gene expression levels in *P. aeruginosa* by 98.09 and 64.91 folds, respectively. They noticed that treatment of the isolates with CH/ZnO nanocomposite greatly reduced *LasI* and *RhlI* gene expression compared to chitosan only. When chitosan is combined with nanomaterials, it develops a higher surface-to-volume ratio; the large surface area of CS-NPs enables it to be tightly absorbed to the surface of bacteria, thereby disrupting the membrane leading to leakage of intracellular compounds and subsequently cell death [44],[45].

## 5. Conclusions

The application of CS-NPs reduces the virulence factors significantly at both genotypic and phenotypic levels. These promising results can breathe hope in the fight against resistant *P. aeruginosa* by repressing its QS-regulated virulence factors.
